# Supplementary material for: Pyruvate Dehydrogenase Kinase 1 inhibition mediated oxidative phosphorylation enhancement in cartilage promotes osteoarthritis progression
Source: BMC Musculoskelet Disord. 2023 Jul 20;24:597. doi: 10.1186/s12891-023-06585-6 (PMC10357736; doi:10.1186/s12891-023-06585-6)
Supplement: Supplementary file 1 — Additional file 1. [file 12891_2023_6585_MOESM1_ESM.zip › MF.Gsea.1653627316242/GOMF_FLAVIN_ADENINE_DINUCLEOTIDE_BINDING.html]

Details for gene set GOMF\_FLAVIN\_ADENINE\_DINUCLEOTIDE\_BINDING[GSEA]

|  || Dataset | 55457\_collapsed\_to\_symbols.cls.cls #OA\_versus\_normal.cls.cls #OA\_versus\_normal\_repos |
| Phenotype | cls.cls#OA\_versus\_normal\_repos |
| Upregulated in class | OA |
| GeneSet | GOMF\_FLAVIN\_ADENINE\_DINUCLEOTIDE\_BINDING |
| Enrichment Score (ES) | 0.30705968 |
| Normalized Enrichment Score (NES) | 1.3815136 |
| Nominal p-value | 0.036939315 |
| FDR q-value | 0.34425318 |
| FWER p-Value | 1.0 |
Table: GSEA Results Summary

  

Fig 1: Enrichment plot: GOMF\_FLAVIN\_ADENINE\_DINUCLEOTIDE\_BINDING      
 Profile of the Running ES Score & Positions of GeneSet Members on the Rank Ordered List

  

| SYMBOL | TITLE | RANK IN GENE LIST | RANK METRIC SCORE | RUNNING ES | CORE ENRICHMENT || 1 | CYB5R1 | cytochrome b5 reductase 1 [Source:HGNC Symbol;Acc:HGNC:13397] | 30 | 1.052 | 0.0432 | Yes |
| 2 | FMO4 | flavin containing dimethylaniline monoxygenase 4 [Source:HGNC Symbol;Acc:HGNC:3772] | 379 | 0.716 | 0.0470 | Yes |
| 3 | DLD | dihydrolipoamide dehydrogenase [Source:HGNC Symbol;Acc:HGNC:2898] | 565 | 0.635 | 0.0600 | Yes |
| 4 | MMACHC | metabolism of cobalamin associated C [Source:HGNC Symbol;Acc:HGNC:24525] | 642 | 0.608 | 0.0804 | Yes |
| 5 | KMO | kynurenine 3-monooxygenase [Source:HGNC Symbol;Acc:HGNC:6381] | 694 | 0.595 | 0.1022 | Yes |
| 6 | DUS4L | dihydrouridine synthase 4 like [Source:HGNC Symbol;Acc:HGNC:21517] | 862 | 0.552 | 0.1130 | Yes |
| 7 | ILVBL | ilvB acetolactate synthase like [Source:HGNC Symbol;Acc:HGNC:6041] | 898 | 0.543 | 0.1338 | Yes |
| 8 | GFER | "growth factor, augmenter of liver regeneration [Source:HGNC Symbol;Acc:HGNC:4236]" | 1075 | 0.500 | 0.1417 | Yes |
| 9 | PYROXD1 | pyridine nucleotide-disulphide oxidoreductase domain 1 [Source:HGNC Symbol;Acc:HGNC:26162] | 1236 | 0.470 | 0.1496 | Yes |
| 10 | ACAD8 | acyl-CoA dehydrogenase family member 8 [Source:HGNC Symbol;Acc:HGNC:87] | 1319 | 0.455 | 0.1628 | Yes |
| 11 | ACOX1 | acyl-CoA oxidase 1 [Source:HGNC Symbol;Acc:HGNC:119] | 1401 | 0.443 | 0.1757 | Yes |
| 12 | FMO3 | flavin containing dimethylaniline monoxygenase 3 [Source:HGNC Symbol;Acc:HGNC:3771] | 1492 | 0.430 | 0.1873 | Yes |
| 13 | NOX4 | NADPH oxidase 4 [Source:HGNC Symbol;Acc:HGNC:7891] | 1563 | 0.418 | 0.1999 | Yes |
| 14 | NQO2 | N-ribosyldihydronicotinamide:quinone reductase 2 [Source:HGNC Symbol;Acc:HGNC:7856] | 1568 | 0.418 | 0.2177 | Yes |
| 15 | AGPS | alkylglycerone phosphate synthase [Source:HGNC Symbol;Acc:HGNC:327] | 1727 | 0.394 | 0.2224 | Yes |
| 16 | ETFA | electron transfer flavoprotein subunit alpha [Source:HGNC Symbol;Acc:HGNC:3481] | 1797 | 0.385 | 0.2336 | Yes |
| 17 | AIFM1 | apoptosis inducing factor mitochondria associated 1 [Source:HGNC Symbol;Acc:HGNC:8768] | 1838 | 0.380 | 0.2470 | Yes |
| 18 | DDO | D-aspartate oxidase [Source:HGNC Symbol;Acc:HGNC:2727] | 1839 | 0.380 | 0.2634 | Yes |
| 19 | TXNRD3 | thioredoxin reductase 3 [Source:HGNC Symbol;Acc:HGNC:20667] | 2049 | 0.356 | 0.2625 | Yes |
| 20 | MTO1 | mitochondrial tRNA translation optimization 1 [Source:HGNC Symbol;Acc:HGNC:19261] | 2181 | 0.340 | 0.2670 | Yes |
| 21 | MAOB | monoamine oxidase B [Source:HGNC Symbol;Acc:HGNC:6834] | 2192 | 0.339 | 0.2809 | Yes |
| 22 | SQOR | sulfide quinone oxidoreductase [Source:HGNC Symbol;Acc:HGNC:20390] | 2225 | 0.336 | 0.2929 | Yes |
| 23 | SDHA | succinate dehydrogenase complex flavoprotein subunit A [Source:HGNC Symbol;Acc:HGNC:10680] | 2231 | 0.335 | 0.3071 | Yes |
| 24 | CYB5R2 | cytochrome b5 reductase 2 [Source:HGNC Symbol;Acc:HGNC:24376] | 2446 | 0.308 | 0.3037 | No |
| 25 | DHCR24 | 24-dehydrocholesterol reductase [Source:HGNC Symbol;Acc:HGNC:2859] | 3126 | 0.237 | 0.2610 | No |
| 26 | ACOX2 | acyl-CoA oxidase 2 [Source:HGNC Symbol;Acc:HGNC:120] | 3176 | 0.233 | 0.2673 | No |
| 27 | DUS2 | dihydrouridine synthase 2 [Source:HGNC Symbol;Acc:HGNC:26014] | 3182 | 0.233 | 0.2770 | No |
| 28 | ACADSB | acyl-CoA dehydrogenase short/branched chain [Source:HGNC Symbol;Acc:HGNC:91] | 3382 | 0.213 | 0.2707 | No |
| 29 | FMO1 | flavin containing dimethylaniline monoxygenase 1 [Source:HGNC Symbol;Acc:HGNC:3769] | 3666 | 0.189 | 0.2568 | No |
| 30 | ERO1A | endoplasmic reticulum oxidoreductase 1 alpha [Source:HGNC Symbol;Acc:HGNC:13280] | 3686 | 0.187 | 0.2634 | No |
| 31 | FMO5 | flavin containing dimethylaniline monoxygenase 5 [Source:HGNC Symbol;Acc:HGNC:3773] | 3759 | 0.181 | 0.2656 | No |
| 32 | DPYD | dihydropyrimidine dehydrogenase [Source:HGNC Symbol;Acc:HGNC:3012] | 3830 | 0.173 | 0.2676 | No |
| 33 | SQLE | squalene epoxidase [Source:HGNC Symbol;Acc:HGNC:11279] | 4005 | 0.156 | 0.2608 | No |
| 34 | PPOX | protoporphyrinogen oxidase [Source:HGNC Symbol;Acc:HGNC:9280] | 4225 | 0.135 | 0.2495 | No |
| 35 | NOS1 | nitric oxide synthase 1 [Source:HGNC Symbol;Acc:HGNC:7872] | 4228 | 0.134 | 0.2552 | No |
| 36 | NOX5 | NADPH oxidase 5 [Source:HGNC Symbol;Acc:HGNC:14874] | 4263 | 0.131 | 0.2582 | No |
| 37 | MTHFR | methylenetetrahydrofolate reductase [Source:HGNC Symbol;Acc:HGNC:7436] | 4334 | 0.125 | 0.2582 | No |
| 38 | GSR | glutathione-disulfide reductase [Source:HGNC Symbol;Acc:HGNC:4623] | 4597 | 0.105 | 0.2423 | No |
| 39 | IVD | isovaleryl-CoA dehydrogenase [Source:HGNC Symbol;Acc:HGNC:6186] | 4950 | 0.076 | 0.2181 | No |
| 40 | ACADM | acyl-CoA dehydrogenase medium chain [Source:HGNC Symbol;Acc:HGNC:89] | 4983 | 0.074 | 0.2188 | No |
| 41 | CYB5R3 | cytochrome b5 reductase 3 [Source:HGNC Symbol;Acc:HGNC:2873] | 4986 | 0.074 | 0.2219 | No |
| 42 | QSOX1 | quiescin sulfhydryl oxidase 1 [Source:HGNC Symbol;Acc:HGNC:9756] | 6130 | -0.015 | 0.1334 | No |
| 43 | AOX1 | aldehyde oxidase 1 [Source:HGNC Symbol;Acc:HGNC:553] | 6243 | -0.024 | 0.1257 | No |
| 44 | DAO | D-amino acid oxidase [Source:HGNC Symbol;Acc:HGNC:2671] | 6252 | -0.024 | 0.1261 | No |
| 45 | ACADVL | acyl-CoA dehydrogenase very long chain [Source:HGNC Symbol;Acc:HGNC:92] | 6394 | -0.034 | 0.1166 | No |
| 46 | ACADL | acyl-CoA dehydrogenase long chain [Source:HGNC Symbol;Acc:HGNC:88] | 6427 | -0.037 | 0.1157 | No |
| 47 | CYBB | cytochrome b-245 beta chain [Source:HGNC Symbol;Acc:HGNC:2578] | 6610 | -0.050 | 0.1037 | No |
| 48 | ACOX3 | "acyl-CoA oxidase 3, pristanoyl [Source:HGNC Symbol;Acc:HGNC:121]" | 6747 | -0.059 | 0.0957 | No |
| 49 | TXNRD1 | thioredoxin reductase 1 [Source:HGNC Symbol;Acc:HGNC:12437] | 6820 | -0.064 | 0.0928 | No |
| 50 | PRODH | proline dehydrogenase 1 [Source:HGNC Symbol;Acc:HGNC:9453] | 6927 | -0.074 | 0.0878 | No |
| 51 | COQ6 | "coenzyme Q6, monooxygenase [Source:HGNC Symbol;Acc:HGNC:20233]" | 7156 | -0.092 | 0.0740 | No |
| 52 | MICAL2 | "microtubule associated monooxygenase, calponin and LIM domain containing 2 [Source:HGNC Symbol;Acc:HGNC:24693]" | 7378 | -0.109 | 0.0614 | No |
| 53 | FOXRED2 | FAD dependent oxidoreductase domain containing 2 [Source:HGNC Symbol;Acc:HGNC:26264] | 7514 | -0.120 | 0.0561 | No |
| 54 | ERO1B | endoplasmic reticulum oxidoreductase 1 beta [Source:HGNC Symbol;Acc:HGNC:14355] | 7954 | -0.152 | 0.0284 | No |
| 55 | NDOR1 | NADPH dependent diflavin oxidoreductase 1 [Source:HGNC Symbol;Acc:HGNC:29838] | 7982 | -0.154 | 0.0330 | No |
| 56 | MTRR | 5-methyltetrahydrofolate-homocysteine methyltransferase reductase [Source:HGNC Symbol;Acc:HGNC:7473] | 8087 | -0.162 | 0.0319 | No |
| 57 | GCDH | glutaryl-CoA dehydrogenase [Source:HGNC Symbol;Acc:HGNC:4189] | 8180 | -0.170 | 0.0320 | No |
| 58 | MAOA | monoamine oxidase A [Source:HGNC Symbol;Acc:HGNC:6833] | 8271 | -0.177 | 0.0327 | No |
| 59 | XDH | xanthine dehydrogenase [Source:HGNC Symbol;Acc:HGNC:12805] | 8366 | -0.183 | 0.0333 | No |
| 60 | ACOXL | acyl-CoA oxidase like [Source:HGNC Symbol;Acc:HGNC:25621] | 8377 | -0.184 | 0.0404 | No |
| 61 | CRY2 | cryptochrome circadian regulator 2 [Source:HGNC Symbol;Acc:HGNC:2385] | 8536 | -0.196 | 0.0366 | No |
| 62 | KDM1A | lysine demethylase 1A [Source:HGNC Symbol;Acc:HGNC:29079] | 8608 | -0.202 | 0.0398 | No |
| 63 | ACADS | acyl-CoA dehydrogenase short chain [Source:HGNC Symbol;Acc:HGNC:90] | 9105 | -0.240 | 0.0115 | No |
| 64 | FMO2 | flavin containing dimethylaniline monoxygenase 2 [Source:HGNC Symbol;Acc:HGNC:3770] | 9871 | -0.309 | -0.0348 | No |
| 65 | ACAD10 | acyl-CoA dehydrogenase family member 10 [Source:HGNC Symbol;Acc:HGNC:21597] | 9909 | -0.313 | -0.0242 | No |
| 66 | TXNRD2 | thioredoxin reductase 2 [Source:HGNC Symbol;Acc:HGNC:18155] | 9922 | -0.314 | -0.0115 | No |
| 67 | NOS3 | nitric oxide synthase 3 [Source:HGNC Symbol;Acc:HGNC:7876] | 9980 | -0.319 | -0.0022 | No |
| 68 | POR | cytochrome p450 oxidoreductase [Source:HGNC Symbol;Acc:HGNC:9208] | 10178 | -0.338 | -0.0029 | No |
| 69 | ETFDH | electron transfer flavoprotein dehydrogenase [Source:HGNC Symbol;Acc:HGNC:3483] | 10203 | -0.341 | 0.0100 | No |
| 70 | CRY1 | cryptochrome circadian regulator 1 [Source:HGNC Symbol;Acc:HGNC:2384] | 11247 | -0.463 | -0.0513 | No |
| 71 | STEAP4 | STEAP4 metalloreductase [Source:HGNC Symbol;Acc:HGNC:21923] | 11340 | -0.477 | -0.0378 | No |
| 72 | MICAL3 | "microtubule associated monooxygenase, calponin and LIM domain containing 3 [Source:HGNC Symbol;Acc:HGNC:24694]" | 11638 | -0.523 | -0.0384 | No |
| 73 | NOS2 | nitric oxide synthase 2 [Source:HGNC Symbol;Acc:HGNC:7873] | 12262 | -0.665 | -0.0582 | No |
| 74 | DUS1L | dihydrouridine synthase 1 like [Source:HGNC Symbol;Acc:HGNC:30086] | 12355 | -0.693 | -0.0354 | No |
| 75 | MICAL1 | "microtubule associated monooxygenase, calponin and LIM domain containing 1 [Source:HGNC Symbol;Acc:HGNC:20619]" | 12483 | -0.742 | -0.0132 | No |
| 76 | PRODH2 | proline dehydrogenase 2 [Source:HGNC Symbol;Acc:HGNC:17325] | 12847 | -1.055 | 0.0041 | No |
Table: GSEA details [plain text format]

  

Fig 2: GOMF\_FLAVIN\_ADENINE\_DINUCLEOTIDE\_BINDING      
 Blue-Pink O' Gram in the Space of the Analyzed GeneSet

  

Fig 3: GOMF\_FLAVIN\_ADENINE\_DINUCLEOTIDE\_BINDING: Random ES distribution      
 Gene set null distribution of ES for **GOMF\_FLAVIN\_ADENINE\_DINUCLEOTIDE\_BINDING**
